# Skin Antiageing and Systemic Redox Effects of Supplementation with Marine Collagen Peptides and Plant-Derived Antioxidants: A Single-Blind Case-Control Clinical Study

**DOI:** 10.1155/2016/4389410

**Published:** 2016-01-19

**Authors:** Chiara De Luca, Elena V. Mikhal'chik, Maxim V. Suprun, Michael Papacharalambous, Arseniy I. Truhanov, Liudmila G. Korkina

**Affiliations:** ^1^Evidence-Based Well-Being Ltd., 31 Alt-Stralau, 10245 Berlin, Germany; ^2^Russian Institute of Physical Chemical Medicine, 1A Malaya Pirogovskaya Street, Moscow 117513, Russia; ^3^Dermatology Clinic “Orthobiotiki Clinical”, 3-5 Sorou Street, 15125 Athens, Greece; ^4^Active Longevity Clinic, Beauty Institute on Arbat, 8 Maliy Nikolopeskovskiy Lane, Moscow 119002, Russia; ^5^Centre of Innovative Biotechnological Investigations (CIBI-Nanolab), 197 Vernadskogo Prospekt, Moscow 119571, Russia

## Abstract

Recently, development and research of nutraceuticals based on marine collagen peptides (MCPs) have been growing due to their high homology with human collagens, safety, bioavailability through gut, and numerous bioactivities. The major concern regarding safety of MCPs intake relates to increased risk of oxidative stress connected with collagen synthesis (likewise in fibrosis) and to ROS production by MCPs-stimulated phagocytes. In this clinical-laboratory study, fish skin MCPs combined with plant-derived skin-targeting antioxidants (AO) (coenzyme Q_10_ + grape-skin extract + luteolin + selenium) were administered to volunteers (*n* = 41). Skin properties (moisture, elasticity, sebum production, and biological age) and ultrasonic markers (epidermal/dermal thickness and acoustic density) were measured thrice (2 months before treatment and before and after cessation of 2-month oral intake). The supplementation remarkably improved skin elasticity, sebum production, and dermal ultrasonic markers. Metabolic data showed significant increase of plasma hydroxyproline and ATP storage in erythrocytes. Redox parameters, GSH/coenzyme Q_10_ content, and GPx/GST activities were unchanged, while NO and MDA were moderately increased within, however, normal range of values.* Conclusions*. A combination of MCPs with skin-targeting AOs could be effective and safe supplement to improve skin properties without risk of oxidative damage.

## 1. Introduction

Dietary vitamins, plant-derived polyphenols, fatty acids, proteins, essential amino acids, and trace elements have demonstrated beneficial effects on skin health and appearance [1–3]; hence, the use of nutraceuticals targeting skin is steadily growing. For example, photoprotection by intake of dietary antioxidants has been a subject of numerous* in vitro*, animal, and human studies [4, 5]. Within this direction, the search for reliable and effective antiageing remedies for both topical and systemic administration has become a “hot spot” for cosmetic, food, and biomedical companies. One of the strongest limitations for skin-targeting plant-derived dietary substances with potential antiageing efficacy is their low bioavailability due to limited and selective penetration through the intestinal barrier, destruction by the intestinal microorganisms, high rate of metabolism, and preferential distribution between tissues and organs [6, 7].

Marine fish collagen and collagen peptides have been widely used as functional foods or dietary supplements due to their homology to human collagen structure [8], safety profile [9], stability, biocompatibility, high bioavailability through gastrointestinal barrier [10], and potent bioactivities [11]. Marine collagen peptides (MCPs) obtained by enzymatic digestion of fish skin have been shown to exert several health effects mainly in two directions: metabolic disorders and skin/bone repair. Thus, they positively affected glucose and lipid metabolism in patients with type II diabetes mellitus [12], improved lipid metabolism in obese people [13] and genetically modified mice [14], ameliorated early alcoholic liver injury [15], and possessed hypotensive and lipid normalising action in patients with primary hypertension [16]. A great majority of publications demonstrated significant wound healing efficacy of orally administered MCPs in animal models of excision and full-thickness skin wounds [10, 17, 18]. Recently, collagen peptides isolated by enzymatic digestion from fish, bovine, and porcine skin as well as from chicken and bovine cartilage have drawn particular interest for the treatment of patients with osteoarthritis. Several clinical trials showed that MCPs were safe and provided an improvement in terms of pain and functions in such patients [19]. From mechanistic point of view, the oral intake of MCPs stimulated the synthesis of extracellular matrix (ECM) macromolecules such as endogenous collagen, by upregulating gene expression of several collagen-modifying enzymes involved in posttranslational collagen modification and cross-linking [20]. Several* in vitro* studies have shown antioxidant properties of very-low-molecular-weight (1–20 Da) MCPs [21, 22] containing proline, which is a scavenger of hydroxyl radicals. Of importance for the present study, MCPs are considered antiageing compounds because they seem to increase life span in rats by inhibiting spontaneous tumour incidence [9], possess photoprotective and immunomodulating properties [23–25], and improve/eliminate signs of premature senescence of human skin [26, 27].

The major concern regarding safety and clinical feasibility of regular intake of MCPs has been raised from the well established fact that the induction of collagen synthesis, mainly assessed by the increased hydroxyproline levels, is often associated with oxidative stress [28–30]. Moreover, MCPs of different origin have been shown to activate innate immune response of macrophages and neutrophils through Toll-like receptor 4, which leads to NADPH-oxidase (NOX4) activation and reactive oxygen species overproduction [31, 32]. A newly developed composition of MCPs with a complex of essential skin-targeting antioxidants, that is, coenzyme Q_10_ + selenium + luteolin + grape-skin extract, demonstrated UVA-protective effects in the preliminary* in vitro* experiments on human skin biopsies [25]. However, the composition under commercial name of CELERGEN^*®*^ has never been evaluated clinically when administered as a food supplement.

The goal of the present clinical-laboratory study was to elucidate the effects of the oral administration of CELERGEN on skin physiology and dermal collagen deposition in the group of healthy middle-aged subjects with clinical signs of skin ageing. The cutaneous clinical-instrumental data were compared with the systemic metabolic parameters of collagen synthesis, redox balance, and energy storage. For the first time, we demonstrated (i) remarkable improvement of ageing skin physiology and structure, which corresponded to enhanced systemic markers of collagen synthesis; (ii) systemic redox balance, sustained by the antioxidant complex; and (iii) increased systemic energy storage. We also hypothesised that moderately increased plasmatic levels of nitric oxide (NO) and malonyl dialdehyde (MDA) may play positive roles of mediators in the MCPs-induced collagen and ATP synthesis/storage, as well as in sebum production. On these grounds, we suggested that selected antioxidants targeting the distinct organs/tissues should be essential components of MCPs-containing nutraceuticals for more effective, individualised, and safe supplementation.

## 2. Materials and Methods

### 2.1. Patients

The study enrolled a group of 41 adult healthy Caucasian volunteers of both sexes recruited from the Beauty Institute on Arbat (Moscow, Russia) staff (age 37–72 years; mean age 50.6 ± 10.4 years; 5 males and 36 females) following the exclusion and inclusion criteria for an open single-blind clinical study. The inclusion criteria were as follows: (i) healthy white adult subjects of both sexes, 35–75 years of age, (ii) subjects with visible symptoms of aged facial skin, (iii) subjects who agreed to interrupt any intake of antioxidant nutraceuticals/drugs for at least 1 week before and during the entire duration of the trial, and (iv) subjects without any difficulty to understand and follow the clinical investigator instructions. Pregnant and breastfeeding women, subjects with allergic/intolerance reactions to any component of the tested product, subjects on any other nutraceutical interventions or/and therapies, and subjects simultaneously engaged in other clinical trials were excluded from the study. The participants were informed that they could interrupt clinical trial at any moment, without any explanation of causative reason for their action, or in case they noticed any adverse reaction to the tested product or had any sensation that the product intake affected their appearance negatively.

The protocol of the clinical trial was duly analysed and approved by the Ethical Committee of the Beauty Institute on Arbat, Moscow, Russia (number 11/EK-2014). All recruited subjects gave their informed consent to personal and anamnestic data collection and biological material sampling. The guidelines of Helsinki Declaration for human experimentation were strictly followed during the conduct of the clinical trial.

### 2.2. Food Supplement under Investigation

Food supplement containing marine collagen peptides derived from skin of deep sea fish (MCPs, 570 mg), grape-skin extract (10 mg), coenzyme Q_10_ of plant origin (10 mg), luteolin (10 mg), and selenium (0.05 mg) of plant origin was formulated in soft gelatine capsules. As inactive solvents, refined and partly hydrogenated soybean oil as well as small admixture of pure soybean lecithin were used. The product, under the commercial name of CELERGEN (manufacturer: Laboratories-Dom, Carouge, Switzerland), was kindly provided by Suisse Ueli Corporation. According to the manufacturer's information, the deep sea fish sources, that is,* Pollachius virens*,* Hippoglossus hippoglossus*, and* Pleuronectes platessa*, originated from the French coast of the North Sea.

Fish skin was homogenised in distilled water, with addition of complex proteases. The enzymatic proteolytic process was carried out at 40°C and pH 8.0 for 3 h, after which the proteases were inactivated by short-term heating (56°C for 10 min). The liquid was sterilised by Millipore filtration (pore size 0.02 mm) and spray-dried to prepare MCP powder, as described in detail previously [17, 33]. Chemical analysis by Kjeldahl assay of the powder confirmed a >90% content of collagen peptides, with moisture and ash content <10%. According to previous publications [10, 33, 34], the molecular weight distribution of MCPs after the described enzymatic digestion process was within the range of 10–60 Da, and MCPs were enriched in glycine, glutamine, proline, hydroxyproline, asparagine, alanine, and arginine.

The aqueous extract of grape skin was obtained from* Vitis vinifera* Linn. fruit and contained at least 70% of polyphenols and 20% of procyanidins as per UV/Vis spectrophotometry data. The coenzyme Q_10_ component of plant origin was of highest purity (100 ± 3%), confirmed by both IR spectrophotometry and high performance liquid chromatography (HPLC) methods. Food-quality luteolin was extracted from Marigold plant petals, and the extract contained 20% of luteolin and 1% of zeaxanthin evaluated by HPLC analysis. Selenium in the form of selenite (according to gravimetric method) was extracted from plant bulbs and leaves. Acute and chronic toxicity data and documents of Certificates of Analyses, Security, and Registration in Switzerland were duly provided by the manufacturer.

### 2.3. Clinical Study Design

The entire trial duration was 4 months (May–December 2014), that is, 2 months of pretreatment period, followed by 2 months of treatment with the test nutraceutical administration. The facial skin parameters of recruited volunteers were analysed three times: at the first visit (enrollment), at the second visit 2 months after the pretreatment period, and at the third visit immediately after the treatment period. Each assessment session comprised instrumental methods for measuring skin physiology parameters and ultrasound properties of the skin layers. This design allowed us to use the same subject as a control and an experiment. During the treatment period, the volunteers were recommended to take 2 capsules of CELERGEN a day (at breakfast and dinner time) for 60 consecutive days. At the second and third visits, the participants donated 20 mL of venous blood after overnight fasting and test tubes were coded by the principal clinical investigator. Blood samples were routinely processed for general haematology (haemoglobin content, differential cell count, and the rate of erythrocyte sedimentation) and biochemistry (glucose levels, plasma protein and lipid profiles, transaminases activities, and C-reactive protein content). Laboratory operators carried out analytical determinations blindly, and statistician was not informed which set of analyses was done in the control or experimental periods, hence ranking this study of clinical efficacy of the nutraceutical as “a single-blind” clinical investigation.

### 2.4. Assessment of Skin Physiology Parameters

Several physiological parameters, mainly barrier properties, of the facial skin were assessed by appropriate SOFT PLUS TOP probes, with microcamera visual analysis and patented computerised programs (Callegari, Parma, Italy). Skin elasticity was determined by the elastometric approach used in the SOFT PLUS technique. The transepidermal water loss (TEWL), an index of skin moisture, was assessed with Tewameter, which measures the water evaporation through cutaneous levels. When the skin is aged or damaged, the barrier properties of the skin are affected, with increased water evaporation and reduced skin hydration. Sebum content was measured by the SOFT PLUS sebometric probe.

### 2.5. Assessment of Ultrasound Properties of the Skin

Assessment of ultrasound properties of the skin was performed by a digital ultrasound imaging system DUB CUTIS (Digital Ultraschall Bildsystem, Germany), which allowed determining four parameters simultaneously: epidermal and dermal thickness and epidermal and dermal ultrasonic density. The first two parameters are indirect markers of collagen (dermis) and lipid (epidermis) synthesis and retention while the second pair of parameters characterises the evenness and order in the epidermal and dermal structures, respectively. The elastic properties of the skin were additionally analysed by a TPM system containing elastometric sensor (22 MHz) which combines digital ultrasound examination with an imaging record (DUB CUTIS, Germany). A computerised multifunctional diagnostic tool integrating different morphometric parameters (epidermal thickness, tone, wrinkles, and elasticity) for face skin biological age determination was used (SOFT PLUS TOP, Callegari, Parma, Italy).

### 2.6. Reagents and Assay Kits

The majority of chemical reagents, HPLC standards, mediums, solvents, and luciferin-luciferase for ATP assay were from Sigma Chemical Co. (St. Louis, MO, USA); kits for enzyme activity assays and Griess reagent for nitrites/nitrates determination were from Cayman Chemical Company (Ann Arbor, MI, USA). Manufacturers of other reagents are mentioned within the respective methods.

### 2.7. Redox and Oxidation Markers' Studies

Complete differential blood cell counts and metabolic analyses were performed on fresh ethylenediaminetetraacetic acid- (EDTA-) anticoagulated venous blood of 12 hrs fasting subjects. Biochemical assays were performed on peripheral blood plasma or red blood cells (RBC), either immediately (ATP, glutathione, and coenzyme Q_10_) or within 72 hrs, on sample aliquots stored at −80°C under argon. Plasma levels of nitrites/nitrates (NO_2_^−^/NO_3_^−^, expressed as *μ*moles/L) were measured spectrophotometrically by Griess reagent [35]. Protein content was measured according to Bradford [36], using a microplate assay kit (Bio-Rad, Hercules, CA, USA). Total glutathione (reduced + oxidized glutathione, GSH + GSSG, mg/g Hb) levels in erythrocytes were measured by HPLC (Shimadzu Scientific Instruments, Columbia, MD, USA) according to Reed et al. [37]. Total coenzyme Q_10_ (CoQ_10_H_2_ + CoQ_10_, mg/L) levels in plasma were quantified by HPLC as described previously [38]. In brief, 1 mL plasma sample, with adequate amount of coenzyme Q_9_ (internal standard) and 500 *μ*L acetic acid (50% solution), was extracted twice, first with 3.5 mL and then with 2.5 mL of ethanol/hexane mixture (2 : 5 vol/vol), with homogenisation and subsequent centrifugation. The upper phase containing hexane extract was evaporated under nitrogen flux and then resuspended in an adjusted amount of a methanol/isopropanol (3 : 2 vol/vol) mixture for HPLC analysis. Reduced and oxidized forms of coenzyme Q_10_ (CoQ_10_H_2_ and CoQ_10_) were quantified simultaneously with HPLC equipped with analytical Supelcosil LP-18 column (24 cm × 4.6 mm, 5 *μ*m, Supelco, Bellefonte, PA, USA) plus its guard column, and in line photodiode array and electrochemical detector (ESA CoulArray, Bedford, MA, USA) in accord with previously published methods [39, 40]. The clinical normality range was extrapolated from the above publications.

Plasmatic Cu,Zn-superoxide dismutase 3 (Cu,Zn-SOD3, U/g protein) activity was measured spectrophotometrically at 505 nm using appropriate kit from Cayman Chemical Company (Ann Arbor, MI, USA) [41, 42]. RBC were lysed in hypotonic solution and the postspin cell lysates were analysed. Total RBC glutathione-S-transferase (GST, U/mg Hb) activity was measured spectrophotometrically by the methods described previously, using chloro-2,3-dinitrobenzene as substrate [43]. RBC glutathione peroxidase (GPx, U/g Hb) activity was determined using Cayman Chemical kit, according to the method [44].

Plasma levels of MDA were determined by slightly modified spectrophotometric analysis of thiobarbituric acid-reactive substances (TBARS) described elsewhere [45]. After a 15 min treatment of plasma (200 *μ*L) with trichloroacetic (1.22 M) and hydrochloric (0.6 M) acids, alkaline solution of TBA was added and the mixture was boiled for 30 min. TBARS were extracted with butanol and analysed spectrophotometrically at 535 nm. The results were expressed in *μ*M of MDA using the appropriate calibration curve.

### 2.8. ATP Measurement in Erythrocytes

100 *μ*L of erythrocyte pellet was stored on ice until analysis. Ice-cold water (990 *μ*L) was added to 10 *μ*L of the erythrocytes pellet and mixed and the lysed erythrocytes were kept on ice. The principle of ATP assay is based on the quantitative bioluminescent determination of* adenosine 5*′*-12 triphosphate (ATP), assessed by the Bioluminescence Assay Kit*. In the assay, ATP is consumed when firefly luciferase catalyses the oxidation of D-luciferin to adenyl-luciferin which, in the presence of oxygen, is converted to oxyluciferin with light emission. This second reaction is essentially irreversible. When ATP is the limiting reagent, the light emitted is proportional to the ATP present. The measurements of luciferin-luciferase chemiluminescence were performed on a Victor2 1420 multilabel counter, equipped with Wallac 1420 Software (Perkin Elmer, MA, USA). Results were expressed as mmoles/L.

### 2.9. Hydroxyproline Assay

The plasma levels of free hydroxyproline (Hyp) and hydroxyproline in the form of oligopeptides, mainly proline-hydroxyproline, were determined by a chemical colorimetric method using a commercial kit (Hydroxyproline Detection Kit) in accord with the manufacturer's instructions. Hyp concentrations were quantified in the linear range of its calibration curve using an array reader (Bio-Rad, Hercules, CA, USA) and expressed in *μ*g/mL of plasma.

### 2.10. Statistical Analysis

Statistical analysis of clinical data was carried out using WINSTAT programs for personal computers (Statistics for Windows 2007, Microsoft, USA). All biochemical and molecular measurements were done in triplicate and data were statistically evaluated. Values were presented as mean, standard error of the mean, and 1.96 × standard error of triplicate analyses. When several datasets were compared, data were analysed by Student's *t*-test for unpaired data. Differences between initial/final data for a single participant were analysed by paired *t*-test and by Mann-Whitney test for changes from baseline. All reported *p* values are from two-tailed tests, and *p* values of less than 0.05 were considered to indicate statistical significance.

## 3. Results

### 3.1. Subjective Evaluation by Participants and Clinical Investigators

All healthy volunteers (*n* = 41) recruited in the trial duly completed it. There were no drop-offs due to low compliance or adverse effects of the supplementation. Routine haematological and biochemical analyses, which were carried out after blood donation in the beginning and after the cessation of the study, did not show statistically significant changes possibly reflecting adverse consequences of the test nutraceutical in the prescribed dosages (data not shown). The subjective evaluation of the product effects on selected general health parameters is shown in Table 1. The participants were predominantly satisfied with the effects obtained on general health conditions and skin properties and partly also by enhanced muscle strength and stamina. No effect whatsoever on digestion was registered.

### 3.2. Effects on Facial Skin Properties

Comparison of digital photos taken before and after clinical trial showed visible qualitative improvement of aesthetic aspect of face with pronounced lifting effect (*data not shown*).

**Figure 1:**
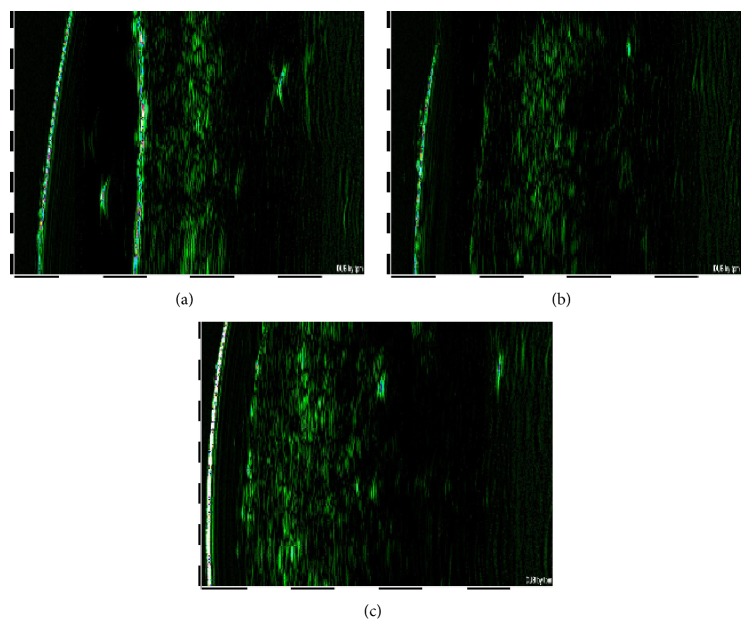
Digital images of facial skin ultrasound examinations (patient number 23, e.g.), made 2 months before the beginning of the trial (a), at the day of the trial beginning (b), and immediately after the trial cessation (c).

Characteristic digital images of ultrasound examinations made at trial beginning (2 months before the beginning of supplementation), at the first day of nutraceutical administration, and immediately after the trial cessation are shown, respectively, in Figures 1(a), 1(b), and 1(c). The individual ultrasonic characteristics were rather stable and were not subjected to statistically significant changes during the 2 months of pretreatment period (Tables 2 and 3; compare columns 1 and 2). The analysis of individual data showed that highly enhanced dermal thickness and homogenous distribution of collagen fibers in dermis were detectable in 23% (*n* = 11) of the participants after the trial cessation. Statistical evaluation of dermal thickness and acoustic density revealed significant changes exclusively at the third visit (Table 2), while the ultrasonic properties of epidermis remained unchanged (Table 3).

Analyses of the main physiological parameters of the skin relevant to ageing, such as elasticity, moisture, and sebum content, demonstrated their comparative stability in the pretreatment period, as there were no significant changes between the first and the second sets of measurements (Table 4, columns 1 and 2).* Conversely*, CELERGEN administration statistically significantly enhanced skin elasticity and sebum production (*p* < 0.0001), whilst not influencing cutaneous moisture (Table 4, columns 2 and 3). Biological age, calculated on the basis of ultrasound and cutaneous physiology measurements, tended to decrease after the trial; however, the difference did not reach statistical significance.

It should be noticed that all tested parameters of skin physiology and structure were not subjected to temporal fluctuations during the 2-month pretreatment period, and therefore changes observed can be viewed as a result of CELERGEN administration.

### 3.3. Plasmatic Oxidation Markers and Antioxidants

**Figure 2:**
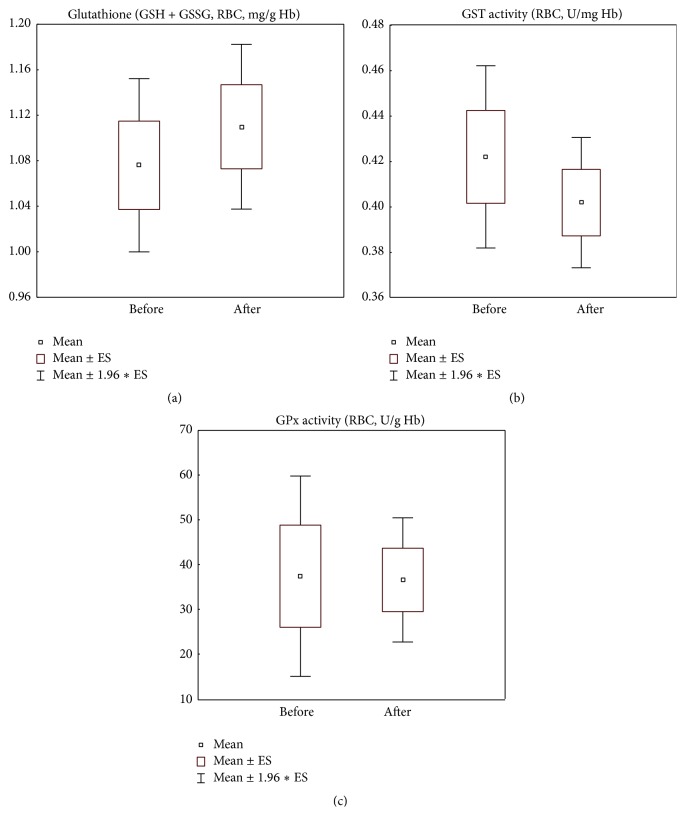
Glutathione cycle parameters: erythrocyte levels of total glutathione (reduced and oxidized forms, GSH + GSSG) (a) and erythrocyte enzymatic activities of glutathione-S-transferase (b) and of glutathione peroxidase (c) in the study group of patients (*n* = 41), before and after food supplement administration period. Values are represented as mean (□), standard error of the mean (upper and lower limits of the box), and 1.96 × standard error (upper and lower whiskers). GSH: reduced glutathione; GSSG: oxidized glutathione; RBC: red blood cells; Hb: haemoglobin; GST: glutathione S-transferase; GPx: glutathione peroxidase. Reference normality range: RBC total glutathione (0.5–1.6 mg/g Hb); RBC GST activity (0.2–0.7 U/mg Hb); RBC GPx activity (18.0–54.0 U/g Hb).

**Figure 3:**
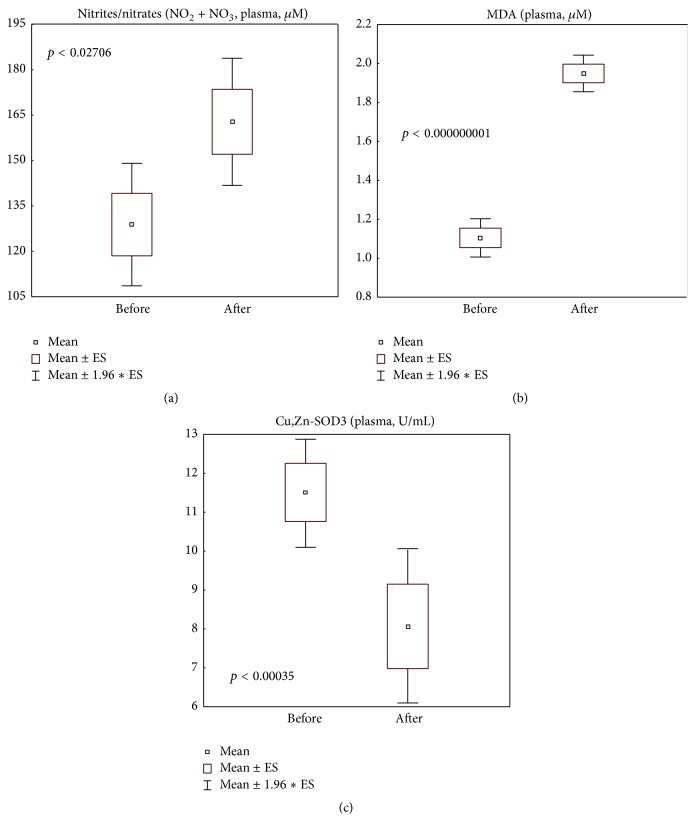
Systemic oxidative stress markers: plasma levels of nitrites/nitrates (NO_2_ + NO_3_) (a), of malonyl dialdehyde (MDA) (b), and of Cu,Zn-superoxide dismutase 3 (Cu,Zn-SOD3) (c) in the study group of patients (*n* = 41), before and after food supplement administration period. Values are represented as mean (□), standard error of the mean (upper and lower limits of the box), and 1.96 × standard error (upper and lower whiskers). Intergroup significant differences (*p*) are indicated in the relative panels. NO_2_ + NO_3_: nitrites + nitrates; MDA: malonyl dialdehyde. Reference normality range: plasma NO_2_ + NO_3_ (70.3–221.0 *μ*M); plasma MDA (1.0–2.2 *μ*M); plasma Cu,Zn-SOD3 (5.0–20.1 U/mL).

Surprisingly, CELERGEN administration did not affect several markers of glutathione metabolism such as total glutathione levels (normality range: 0.5–1.6 mg/g Hb) and glutathione-S-transferase and glutathione peroxidase activities (normality ranges: 0.2–0.7 U/mg Hb and 18.0–54.0 U/g Hb, resp.) (Figures 2(a), 2(b), and 2(c)). At the same time, nitrite/nitrate and MDA levels in plasma (normality ranges: 70.3–221.0 *μ*M and 1.0–2.2 *μ*M, resp.) were statistically significantly increased (*p* < 0.05 and *p* < 0.0001, resp.), although they remained within normal physiological range established in our laboratory (Figures 3(a) and 3(b)). Extracellular Cu,Zn-SOD3 activity was slightly suppressed (*p* < 0.001) but did not drop below the normality border (5.0–20.1 U/mL) (Figure 3(c)).

### 3.4. Parameters of Collagen and ATP Metabolism

**Figure 4:**
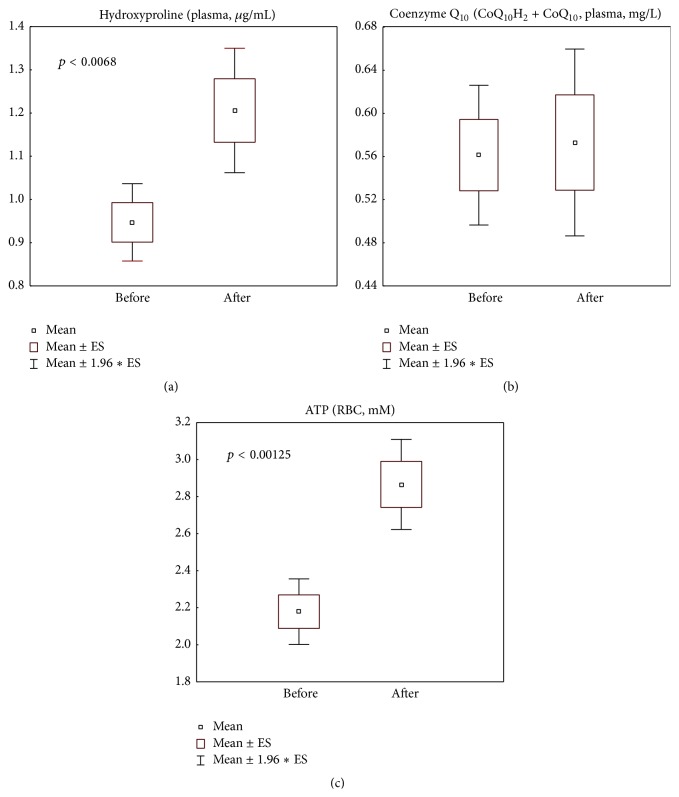
Metabolic parameters related to collagen and ATP synthesis: levels of plasma hydroxyproline (a) and of the lipophilic antioxidant total coenzyme Q_10_ (reduced and oxidized forms, CoQ_10_H_2_ + CoQ_10_) (b) and erythrocyte ATP (c) in the study group of patients (*n* = 41), before and after food supplement administration period. Values are represented as mean (□), standard error of the mean (upper and lower limits of the box), and 1.96 × standard error (upper and lower whiskers). Intergroup significant differences (*p*) are indicated in the relative panels. CoQ_10_H_2_: reduced coenzyme Q_10_; CoQ_10_: oxidized coenzyme Q_10_; ATP: adenosine triphosphate; RBC: red blood cells. Reference normality range: total coenzyme Q_10_ (0.4–1.6 mg/L); ATP (1.0–4.0 mM).

Plasma content of hydroxyproline was found highly elevated (*p* < 0.01) (Figure 4(a)), the same with ATP content in erythrocytes (*p* < 0.001) (Figure 4(c)), although total content of coenzyme Q_10_ was not changed after supplementation with the coenzyme Q_10_-containing nutraceutical (Figure 4(b)).

## 4. Discussion

In a preliminary* ex vivo* study of CELERGEN components against UVA-induced damage in human skin biopsies and fibroblasts [25], marine collagen peptides but not the complex of plant-derived antioxidants inhibited transcriptional and posttranscriptional matrix metalloproteinase-1 and elastase upregulation, leading the authors to hypothesise clinical feasibility for the prevention of skin photoaging. In contrast, another publication demonstrated that the bioflavonoid luteolin, a component of the CELERGEN antioxidant complex, effectively attenuated UVB-induced DNA damage, inflammation, and ROS overproduction in skin cells* in vitro* and* in vivo* [46].

In the present study, we obtained convincing clinical data on the efficacy of the marine collagen peptide and plant antioxidant formulation CELERGEN in improving dermal collagen deposition and structure (Table 2), as well as skin elasticity (Table 4). These effects were consistent with enhanced plasma levels of hydroxyproline, a systemic metabolic marker of collagen synthesis (Figure 4(a)). Nearly 100% of human Hyp is in fact found in collagen [47]. Hyp being an oxidative derivative of proline, both amino acids are essential for collagen biosynthesis, maturation, mode of deposition, and collagen fiber structure. Dietary proline intake promotes tissue repair in humans and animals [48]. Recently, Wang et al. [17] reported the experimental evidence that MCPs might improve collagen synthesis and maturation by inducing the expression of transforming growth factor beta-1 (TGF-*β*1) and basic fibroblast growth factor (bFGF). Our data (Figure 4(a)) are consistent with previously published ones on rats fed with MCPs from salmon or trout skin [49], showing that plasma levels of free and dipeptide (Pro-Hyp) forms of hydroxyproline were highly increased after single intake of MCPs in soybean oil. Similar data on the blood levels of Hyp and Hyp-containing peptides were obtained on healthy human volunteers [50].

Numerous animal studies on the effects of oral administration of natural or synthetic antioxidants towards collagen deposition, reactive species levels, and antioxidant defences generated highly conflicting data, depending on the experimental system. Thus, with various wound healing models, it was repeatedly demonstrated that either complex plant extracts containing active secondary metabolites (triterpenes, polyphenols, alkaloids, etc.) [18, 51] or a composition of collagen inducing polysaccharides like chitosan and antioxidants such as curcumin [52] or resveratrol [53] ameliorated wound healing increasing skin collagen deposition, while suppressing proinflammatory iNOS and myeloperoxidase, decreasing pathologically elevated levels of MDA and hydrogen peroxide, and improving enzymatic antioxidant defence. Recent studies showed that collagen peptides from fish skin remarkably promoted both wound healing and angiogenesis in different experimental settings [10, 17]. Of importance, excessive NO produced during the inflammatory phase of wound healing process impaired collagen accumulation [54], while moderate NO levels accelerated the granulation phase of wound closure [18, 55]. Moreover, wound healing acceleration by moderate levels of H_2_O_2_ through induction of vascular endothelial growth factor in keratinocytes and macrophages was proved in a number of experimental and clinical studies [56, 57]. Here, we found that, along with Hyp accumulation, plasma levels of nitrites and nitrates, related to NO production in the bloodstream, were moderately increased after CELERGEN treatment, though remaining within the range of normal values (Figure 3(a)). Similar results were obtained with plasmatic MDA (Figure 3(b)). This allowed us to suggest that redox regulation of cutaneous collagen synthesis process or/and fibroblast proliferation activation could have occurred due to physiologically relevant NO and/or MDA amounts generated following supplement intake. However, the suggestion deserves further mechanistic* in vitro* and clinical research.

On the other hand, in the models of cardiac fibrosis [58, 59], the significant decrease of the model-related oxidative stress obtained by the use of* Momordica charantia* fruit extract [58] or* Fructose Chorpondiatis* total flavonoids was indeed associated with simultaneous attenuation of collagen deposition, as assessed by Hyp levels. Similar results were obtained in other tissue models of fibrosis [28, 60–62], including skin fibrosis [29]. It seems that complex mixtures of fruit extracts contained both collagen synthesis affecting agents and antioxidants.

UV irradiation could cause skin photodamage causing the symptoms of premature photoageing. Evaluating the photoprotective effects of dietary MCPs isolated from jellyfish umbrella [24] or from fish scale [63] in the model of chronic UVA + UVB irradiation of mice, the authors concluded that MCPs enhanced skin immunity, reduced water loss, restored cutaneous collagen and elastin levels and structure, and maintained type III to I collagen ratio. Under similar experimental design, Zhuang et al. [64] showed the protective action of MCPs on antioxidant enzymes activities and glutathione, lipid, and Hyp contents of murine skin. In this connection, we found a significant reduction (within the range of normality) of plasmatic SOD3 activity following CELERGEN supplementation (Figure 3(c)). Extracellular plasmatic Cu,Zn-SOD3, a glycoprotein with a heparin-binding domain, is predominantly expressed in tissue ECM, where it is bound to heparin sulfate proteoglycan [65]. Physiologically, SOD3 maintains redox balance and tissue homeostasis and modulates innate and adaptive immune responses. Cutaneous homeostasis strongly depends on the ECM microenvironment; therefore, an elevated SOD3 activity may be a marker of adaptive response against intrinsic age-associated and external hazardous factors inducing immune suppression in the skin [66].

Since the supplementation of compounds with a direct antioxidant effect has failed so far to show clinical efficacy and sometimes even aggravated clinical picture [67], the search for drugs/therapeutic strategies to modulate oxidative stress has been drastically redirected nowadays towards (1) indirect AOs inducing endogenous enzymatic system of antioxidative defence, mainly, through Nrf2-connected pathway; (2) selective inhibitors of ROS/RNS-producing enzymes, for example, different isoforms of NADPH-oxidase, having shown definite clinical effects; (3) recognising essential and multiple physiological roles of redox balancing agents rather than mere inhibitors of free radical processes. Plant-derived polyphenols, quercetin, resveratrol, luteolin, and many others appear to possess all these multipotent capabilities [6]. The presence of quercetin and resveratrol from grape-skin extract and of luteolin in the antioxidant combination of CELERGEN may then well account for the observed redox balancing effects during the upregulation of MCPs-induced collagen synthesis (Figures 2(a), 2(b), and 2(c)). The majority of publications have in fact demonstrated a drop of GSH content and an increase of protective GPx activity when Hyp content was raised [28, 64, 68]. Of great importance, the presence of antioxidants in the tested formulation, whilst possibly protecting the redox balance from harmful side effects of collagen metabolism, did not negatively affect the desired process of dermal collagen synthesis/deposition. In fact, the observed elevation of plasmatic Hyp was comparable with that found previously [27], with pure fish MCPs in much higher dosages.

In the last decade, endogenously produced systemic and cutaneous redox-active substances (superoxide, hydrogen peroxide, NO, lipid peroxides, stable end products of lipid peroxidation, oxidative metabolites of cholesterol and squalene, etc.), previously recognised exclusively as undesirable metabolic by-products and markers of oxidative damage, have shown essential functions in cellular signalling and regulation of cell proliferation, differentiation, migration, innate immunity, energy production, ECM dynamics, vascular tone, stress responses and adaptation, and inflammation [55, 69–71]. In this frame, the moderate plasmatic elevation of MDA (Figure 3(b)) and NO (Figure 3(a)) observed in this study may reflect the regulatory functions of these mediators both in MCPs-induced collagen synthesis (Figure 4(a)) [69] and in the process of mitochondrial ATP production (Figure 4(c)). Only excessive amounts are damaging as they initiate cell senescence and death. It seems that, notwithstanding coenzyme Q_10_ supplementation during CELERGEN course, its plasmatic levels were not increased (Figure 4(b)), due to elevated consumption of the coenzyme in the mitochondrial cycle of energy production enhancing ATP storage in erythrocytes (Figure 4(c)) and in cell redox balance control (Figures 2(a)–2(c)).

The antiageing effects of CELERGEN supplementation were evidenced also by the highly increased sebum production (Table 4). It is established that the production of sebaceous lipids is strongly age dependent, being low in the prepubertal period, rising with sexual maturation, and gradually declining in the aging populations (starting from 46–55 years) [72] or in UV-induced premature skin ageing [70, 73]. Cutaneous lipid-soluble antioxidants such as vitamin E and squalene decay accordingly [74]. Since skin surface lipids (SSL) play multiple essential roles in skin barrier properties, skin smoothness, elasticity, and moisture, they are regarded as natural guards of normal cutaneous ecology. Moreover, moderate concentrations of specific SSL unsaturated components (squalene, cholesterol, and free fatty acids) are able to generate oxidised lipid by-products (MDA, 4-hydroxynonenal, oxidised cholesterol, and others), since being long recognised as key signalling molecules for skin immune and metabolic responses to environmental insults and microbial invaders [70, 75, 76]. On the other hand, excessive levels of microbially or photooxidised derivatives of unsaturated fatty acids and other sebum lipids could induce a vicious cycle of sebum overproduction followed by oxidation, thus maintaining inflammation characteristic for acne disease [76]. As shown by our clinical data (Table 1), no complaints about skin conditions were registered during the trial. Conversely, the marked improvement of skin elasticity could be attributed not only to collagen deposition in derma, but also to a moderate physiological increase of SSL content.

**Figure 5:**
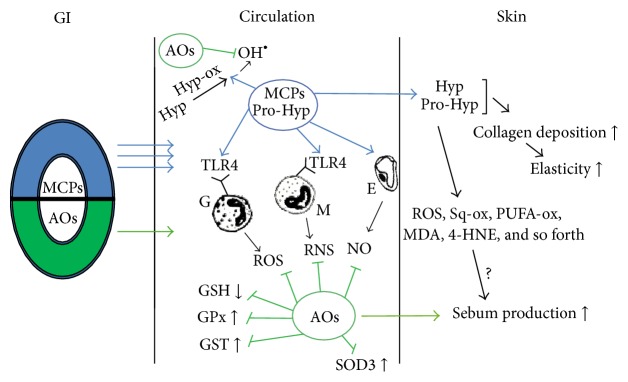
Scheme of the hypothesised redox-dependent mechanisms of CELERGEN physiological effects. Marine collagen peptides (MCPs) easily penetrate gastrointestinal wall (GI, three arrows) and through blood circulation are mainly deposited in the skin. Antioxidant component of the nutraceutical is partly metabolised in GI thus possessing low bioavailability (one arrow); however, skin-targeting antioxidants and their metabolites reach different skin layers. While in the circulation, MCPs stimulate blood phagocytes (granulocytes and monocytes) and endotheliocytes (E) to produce reactive oxygen species (ROS) and reactive nitrogen species (RNS) by activating Toll-like receptors 4 (TLR4). Hydroxyproline (HYP) and prolyl-hydroxyproline (Pro-HYP) dipeptides as major components of MCPs are metabolised by corresponding oxidases and hydroxyl radicals are formed as by-products. Antioxidants prevent systemic oxidative stress blocking GSH oxidation, GPx, GST, and SOD3 activation. In the skin, collagen synthesis and deposition as well as elasticity are increased while (hypothetically) low levels of oxidised forms of skin lipids such as unsaturated fatty acids (PUFA-ox), squalene (Sq-ox), malonyl dialdehyde (MDA), and 4-hydroxy-2-nonenal (4-HNE) may facilitate cell signalling for ATP synthesis and sebum production.

On the grounds of the results obtained and existing literature data, we hypothesised redox-dependent pathways (Figure 5) which may lead to clinical and generalised health effects of CELERGEN supplementation. Obviously, more profound basic research and further clinical studies are needed to prove this hypothesis and to evaluate the underlying mechanisms.

## 5. Conclusions

The addition of dietary plant-derived antioxidants with known skin tropism and health effects towards human skin did not impair definite induction of collagen synthesis and its deposition as compact organised fibres in the dermal layer by marine fish skin-derived collagen peptides. Additional beneficial effects of antioxidants were observed systemically, as normal balance of systemic endogenous antioxidant defence was maintained, and protection of energy storage occurred.

## Figures and Tables

**Table 1:** Subjective evaluation of the 2-month food supplement administration effects, by participants (*n* = 41).

Parameter	Number (%) of participants		
	Improvement	No effect	Aggravation
General health conditions	21 (51%)	20 (49%)	0 (0%)
Stamina/muscle strength/joint motility	15 (36%)	26 (64%)	0 (0%)
Digestive system	0 (0%)	41 (100%)	0 (0%)
Skin conditions	25 (61%)	16 (39%)	0 (0%)

**Table 2:** Effects of the 2-month food supplement administration on the ultrasonic properties of the dermis (*n* = 41).

Parameter	Dermis		
	Pretreatment period	Before treatment	After treatment
Thickness, *μ*m	3884 ± 30	3900 ± 31	4133 ± 28^*∗*^
Acoustic density	5.2 ± 0.2	5.1 ± 0.2	6.3 ± 0.1^*∗*^

^*∗*^ *p* < 0.05  *versus* “before treatment.”

**Table 3:** Effects of the 2-month food supplement administration on the ultrasonic properties of the epidermis (*n* = 41).

Parameter	Epidermis		
	Pretreatment period	Before treatment	After treatment
Thickness, *μ*m	76.9 ± 1.0	77.0 ± 0.8	77.6 ± 0.9
Acoustic density	35.6 ± 2.4	35.2 ± 2.2	35.4 ± 2.0

**Table 4:** Effects of the 2-month food supplement administration on the parameters of skin physiology (*n* = 41).

Parameter	Pretreatment period	Before treatment	After treatment
Elasticity	34.06 ± 1.54	33.66 ± 1.21	40.26 ± 0.87^*∗∗∗*^
Moisture	48.83 ± 3.02	49.03 ± 3.52	46.54 ± 3.02
Sebum	29.89 ± 4.16	29.37 ± 4.76	56.86 ± 4.04^*∗∗∗*^
Skin biological age	50.11 ± 1.91	49.51 ± 1.68	48.09 ± 1.74

^*∗∗∗*^ *p* < 0.0001  *versus* “before treatment.”

## Abbreviations

MCPs: Marine collagen peptides
ECM: Extracellular matrix
NOX4: NADPH-oxidase
NO: Nitric oxide
MDA: Malonyl dialdehyde
HPLC: High performance liquid chromatography
TEWL: Transepidermal water loss
EDTA: Ethylenediaminetetraacetic acid
RBC: Red blood cells
Cu,Zn-SOD3: Cu,Zn-superoxide dismutase 3
GST: Glutathione-S-transferase
GPx: Glutathione peroxidase
TBARS: Thiobarbituric acid-reactive substances
Hyp: Hydroxyproline
TGF-*β*1: Transforming growth factor beta-1
bFGF: Basic fibroblast growth factor
ROS: Reactive oxygen species
RNS: Reactive nitrogen species.
